# Larynx carcinoma regulates tumor-associated macrophages through PLGF signaling

**DOI:** 10.1038/srep10071

**Published:** 2015-05-11

**Authors:** Xu Zhou, Ying Qi

**Affiliations:** 1 Otorhinolaryngology Department, Zhongshan Hospital, Fu Dan University, 180 Fenglin Road, Shanghai 200032, China; 2 State Key Laboratory of Molecular Engineering of Polymers (Fudan University), 220 Handan Road, Shanghai 200433, China

## Abstract

Cancer neovascularization plays an essential role in the metastasis of larynx carcinoma (LC). However, the underlying molecular mechanisms are not completely understood. Recently, we reported that placental growth factor (PLGF) regulates expression of matrix metalloproteinase 3 (MMP3) through ERK/MAPK signaling pathway in LC. Here, we show that MMP9 upregulated in LC, and appeared to be mainly produced by M2 macrophages (tumor-associated macrophages (TAM)). In a transwell co-culture system, PLGF secreted by LC cells triggered macrophage polarization to a TAM subtype that releases MMP9. Moreover, MMP9 was found to be activated in the PLGF-polarized TAM via transforming growth factor β (TGFβ) receptor signaling activation. Furthermore, PLGF in LC cells induced macrophage polarization *in vivo*, and significantly promoted the growth of LC. Thus, together with our previous work, our study highlights a pivotal role of cross-talk between TAM and LC in regulating the metastasis of LC.

A large number of diagnosed larynx carcinoma (LC) have reached advanced stages with metastasis, largely due to being asymptomatic or non-specifically symptomatic of LC in the early stages^1^. These features of LC specifically highlight the importance of understanding the molecular mechanisms underlying the regulation of the metastasis of LC. However, our current knowledge is far from complete.

Neo-angiogenesis is essential for cancer survival, invasion and metastasis of LC^2^,^3^,^4^. Cancer cells secrete proteinases to break through extracellular matrix^5^. The matrix metalloproteinase (MMP) family members are the most important proteinases responsible for the breakdown of extracellular matrix in normal physiological processes and in disease processes, such as cancer metastasis^6^,^7^. MMP9 is a member of MMP family, and overexpression of MMP9 has been reported in various cancer in that it facilitates metastatic spread of different cancer cells^8^,^9^,^10^,^11^,^12^, and essentially LC^13^. Nevertheless, how MMP9 is regulated in LC has not been studied yet.

Moreover, cancer cells also produce angiogenic factors to induce formation of new vessels^14^,^15^. During these processes, vascular endothelial growth factor (VEGF) family molecules (VEGF-A, VEGF-B, VEGF-C, VEGF-D, VEGF-E and placental growth factor (PLGF)) appear to play critical roles^16^,^17^,^18^,^19^,^20^,^21^,^22^. Among all these VEGF family members, PLGF is specifically involved in pathological vessel formation, whereas its effects could be cancer-dependent^23^,^24^. VEGF receptor I (VEGFR1) is the unique receptor for PLGF, and is expressed on the surface of endothelial cells and monocyte/macrophages.

Macrophages are white blood cells that engulf and digest cellular debris, foreign substances, microbes, and neoplastic cells^25^,^26^,^27^,^28^. Besides these macrophages with a classical phenotype termed “M1” macrophages, another macrophage phenotype termed “M2” mediates humoral immunity and tissue repair^25^,^26^,^27^,^28^. A given macrophage can shift between M1 or M2 phenotype, which is termed “polarization”^25^,^26^,^27^,^28^. The M1 macrophages express nitric oxide synthase (iNOS) and some proinflammatory cytokines and toxic mediators like reactive oxygen species (ROS)^25^,^26^,^27^,^28^. M2 macrophages are specified as expression of CD163, CD206, arginase and CD301^25^,^26^,^27^,^28^. Specifically, M2 macrophages are also called tumor-associated macrophages (TAM), since they secrete a wide range of chemokines, enzymes and growth factors to promote neovascularization, growth and metastasis of cancer cells^29^. Recently, M2 macrophages have been shown to secret extremely high levels of transforming growth factor β1 (TGFβ1) in a pancreas injury model, and the downstream TGFβ receptor signaling can be efficiently inhibited by a TGFβ receptor I inhibitor SB431542^30^.

Recently, we reported that PLGF regulates expression of MMP3 through ERK/MAPK signaling pathway in LC cells^31^. Here, we found that MMP9 also upregulated in LC, but were mainly produced by TAM, rather than by LC cells themselves. In a transwell co-culture system, we found that PLGF secreted by LC cells triggered the macrophage polarization to a TAM subtype, which subsequently activated TGFβ receptor signaling to produce MMP9. Moreover, PLGF was essential for induction of macrophage polarization *in vivo*, and substantially promoted the growth of LC.

## Materials and Methods

### Patient tissue specimens

In the current study, a total of 43 resected LC specimens were investigated. All specimens had been histologically and clinically diagnosed at the Otorhinolaryngology Department of Zhongshan Hospital of Fudan University from 2007 to 2014. The paired resected LC specimen and the adjacent normal tissue (NT) from the same patient were analyzed and compared with each other. The methods for using these clinical materials were carried out in accordance with the approved guidelines from the IACUC of Zhongshan Hospital of Fudan University, with prior patient’s consents.

### Cell lines, transfection and reagents

Hep2 is a human larynx carcinoma line purchased from American Type Cell Culture (ATCC, Rockville, MD, USA), and was cultured in Dulbecco’s modified eagle’s media (DMEM) supplemented with 15% fetal bovine serum (Invitrogen, Carlsbad, CA, USA), as has been described before^31^. Hep2 cells were transfected either with a PLGF-overexpressing plasmid (PLGF), or a small short hairpin interfering RNA for PLGF (shPLGF), or empty plasmid as a control, as has been previously described^31^. The plasmids also contain a GFP reporter for selection of positive cells, and a luciferase reporter for *in vivo* tracing. Inhibitor SB431542 was purchased from Abcam (Cambridge, MA, USA), and used according to the introduction from the manufacturer. Recombinant PLGF was purchased from Sigma-Aldrich (St. Louis, MO, USA). Anti-VEGFR1 antibody was purchased from R&D Biosystem (Los Angeles, CA, USA).

### Animal manipulation

All mouse experiments were performed in accordance with the approved guidelines from the IACUC of Zhongshan Hospital of Fudan University. Male C57BL/6 mice (Jackson Lab, Bar Harbor, ME, USA) of 12 weeks of age were used for isolation of bone marrow-derived macrophages. Female NOD/SICD mice (Jackson Lab) of 12 weeks of age were used for *in vivo* implantation of LC cells. For generation of LC implantation model, 10^6^ luciferase-carrying PLGF-modified Hep2 cells were subcutaneously injected into the tissue close to larynx in NOD/SCID mice, as has been described before^32^,^33^. The tumor growth was monitored and quantified by luminescence levels. Bioluminescence was measured with the IVIS imaging system (Xenogen Corp., Alameda, CA, USA). All of the images were taken 10 minutes after intraperitoneal injection of luciferin (Sigma-Aldrich) of 150 mg/kg body weight, as a 60-second acquisition and 10 of binning. During image acquisition, mice were sedated continuously via inhalation of 3% isoflurane. Image analysis and bioluminescent quantification was performed using Living Image software (Xenogen Corp.).

### Tumor digestion and cell sorting by flow cytometry

LC samples from patients or mice were removed, minced into small pieces, and digested in the digestion media containing 40 mg/dl collagenase (Sigma, USA) and 0.05% trypsine (Sigma-Aldrich) at 37 °C for 30 min. After the digestion, the cells that passed a 40 μm filter were pelleted and re-suspended in physiological solution, labeled with specific antibodies, and then used for flow cytometric analysis. Macrophages were analyzed by flow cytometry with specific fluorescence-conjugated antibodies (F4/80 and CD206--a M2 macrophage marker--, all from Becton-Dickinson Biosciences, San Jose, CA, USA). Data were collected on a FACSCalibur (Becton-Dickinson Biosciences) and analyzed using FlowJo software (Flowjo LLC, Ashland, OR, USA). The percentage expression of each marker on macrophages was determined by the percentage of positive events, as determined by the isotype-matched negative control.

### Generation of bone marrow-derived macrophages

Femurs and tibiae were harvested from 12-week-old male C57BL/6 mice and the bones were flushed with macrophage culture media (high-glucose DMEM supplemented with 10% fetal bovine serum, 10% L929 conditioned media (containing M-CSF, which is essential for macrophage culture), 2% MEM nonessential amino acids, 1% l-glutamine, 1% HEPES, 1% penicillin-streptomycin and 0.1% 2-mercaptoethanol) using a 26-gauge needle into a 50 ml conical tube. The collected bone marrow was centrifuged at 1200 RPM for 5 minutes at 4 °C. The pellets were re-suspended and seeded at 10^6^ per well in 24-well plates and cultured in a 37 °C humid CO_2_ (5%) incubator. The cells were fed with fresh media every other day for 7 days, after which the cells were replenished with L929-free media for co-culture study.

### Transwell co-culture system

Isolated bone-marrow-derived macrophages (10^6^) were co-cultured either with Hep2-shPLGF (10^5^), or with Hep2-Null (10^5^), or with Hep2-PLGF (10^5^) with/without of 10 μmol/l SB431542, or with recombinant PLGF (100 ng) with/without of 10 μmol/l SB431542, as has been described before^30^. Two days after co-culture, macrophages were isolated for flow cytometry and for the subsequent RNA analyses. The conditioned media were used for ELISA analyses.

### RT-qPCR

RT-qPCR was performed as has been described before^31^. All primers (β-actin, MMP9 and TGFβ1) were purchased from Qiagen (Hilden, Germany). Values of genes were normalized against β-actin and then compared with control.

### Western blot

The protein was extracted from the cultured cells. Primary antibodies were anti-SMAD3 (total SMAD3) and anti-phosphorylated SMAD3 (pSMAD3) (all purchased from Cell Signaling, San Jose, CA, USA). Secondary antibodies were HRP-conjugated anti-rabbit, and were all purchased from Jackson ImmunoResearch Labs (West Grove, PA, USA).

### ELISA assay

ELISA was performed as has been described before^31^, using a MMP9 or TGFβ1 ELISA Kit (R&D System, Los Angeles, CA, USA).λ

### Immunohistochemistry

Mouse implanted tumors were dissected out and fixed with 4% paraformaldehyde (Sigma-Aldrich) for 6 hours, and then cyro-protected in 30% sucrose for 24 hours. Frozen samples were then sectioned in 6 μm. Primary antibodies used in immunohistochemistry are rat polyclonal anti-mouse Ki-67 (1:300) (Abcam, Cambridge, MA, USA).

### Statistical analysis

Statistical analyses were performed with SPSS 17.0 statistical software, as has been described before^31^. Data were statistically analyzed using one-way ANOVA with a Bonferoni Correction. Fisher’s exact test was used to compare two conditions. All values are depicted as mean ± standard deviation from 5 individuals in each experiment and are considered significant if p < 0.05. Figures were prepared by GraphPad prism 6.0 (GraphPad Software Inc. La Jolla, CA, USA).

## Results

### TAM from LC produced high levels of MMP9 levels in patients

**Figure 1:**
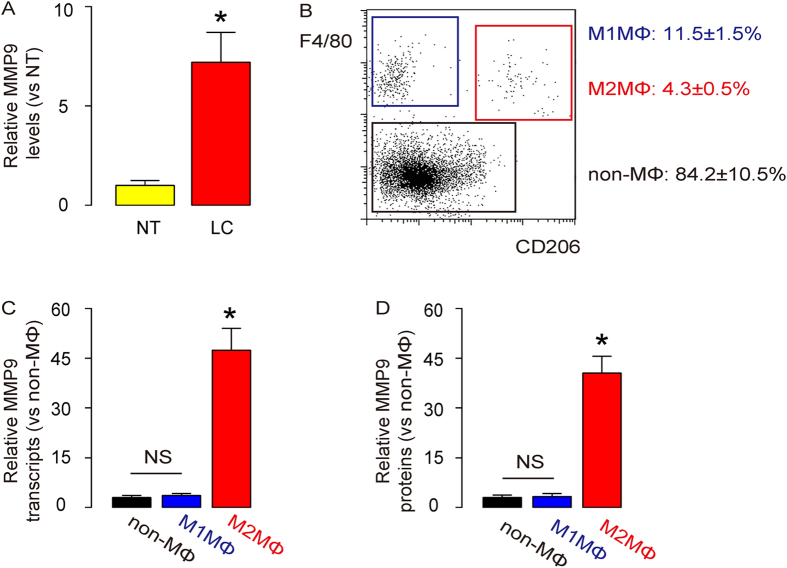
TAM from LC produced high levels of MMP9 levels in patients. (**a**) MMP9 protein levels were examined by ELISA from the resected LC, compared to the adjacent normal tissue (NT) from the patients. (**b**) A representative flow cytometry chart for analyzing digested LC specimen. F4/80, a marker for macrophages. CD206, a marker for M2 macrophages (M2MΦ) or TAM. (**c**) RT-qPCR for MMP9 transcripts in the isolated cells from LC specimen. (**d**) ELISA for MMP9 protein in the isolated cells from LC specimen. M1MΦ: CD206-F4/80 + M1 macrophages (blue rectangle), M2MΦ: CD206 + F4/80 + M2 macrophages (red rectangle), non-MΦ: F4/80- non-macrophages (black rectangle). *: p < 0.05. NS: non-significant. N = 43. Statistic method: one-way ANOVA with a Bonferoni Correction.

We have previously reported a high MMP3 level in LC cells through an autologous PLGF-mediated activation of ERK/MAPK^31^. Besides MMP3, we also detected a significantly higher MMP9 levels in LC, compared to the adjacent normal tissue (NT) from the patients (Fig. 1a). Unlike MMP3 that was previously shown to be produced by LC cells themselves, high MMP9 was mainly detected in a CD206 + F4/80 + population that represents TAM or M2MΦ (Fig. 1b), by RT-qPCR (Fig. 1c), and by ELISA (Fig. 1d), compared to the CD206-F4/80 + M1 macrophages (M1MΦ), and F4/80- non-macrophages (non-MΦ). These data suggest that although both MMP3 and MMP9 may both contribute to the metastasis of LC, their major sources may be different.

### PLGF from LC induced macrophage polarization to a TAM subtype

Since VEGFR1 is the unique receptor for PLGF and VEGFR1 is well-known to be expressed in macrophages to regulate its biology^16^,^17^,^18^,^19^,^20^,^21^,^22^, we hypothesize that PLGF in LC may regulate macrophages in LC tissue and may be responsible for the MMP9 activation in TAM.

**Figure 2:**
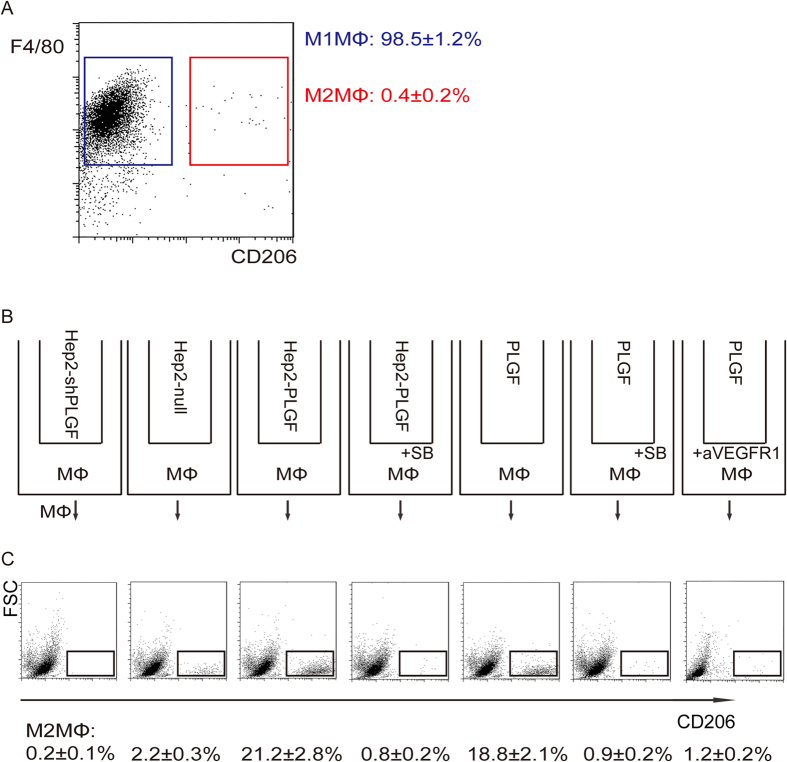
PLGF polarized macrophages to TAM. (**a**) Flow chart for analyzing cultured bone-marrow-derived mouse macrophages. (**b**) Transwell con-culture system. Macrophages were co-cultured with PLGF-adapted Hep2 cells and PLGF alone, with/without SB431542 (SB), or with antibody against VEGFR1 (aVEGFR1). (**c**) M1MΦ and M2MΦ (black rectangle) were analyzed and isolated by flow cytometry based on CD206 (a M2MΦ marker). N = 5.

Then we used a human LC cell line, Hep2, to prove our hypothesis. We have previously shown preparation of PLGF-modified Hep2 cells, Hep2-shPLGF, Hep2-Null and Hep2-PLGF^31^. We isolated macrophages from bone marrow and the phenotype of isolated macrophages was confirmed by flow cytometry (Fig. 2a). Then, we used a co-culture system to examine the effect of these PLGF-modified LC cells on the polarization of bone-marrow derived macrophages and their production of MMP9 (Fig. 2b). macrophages were analyzed for M2MΦ/TAM marker CD206 after two days’ co-culture. We found that macrophages co-cultured with Hep2-shPLGF cells had significantly lower percentage of M2MΦ (0.2 ± 0.1%), compared to macrophages co-cultured with Hep2-Null cells (2.2 ± 0.3%) (p < 0.05, Fig. 2c). On the other hand, macrophages co-cultured with Hep2-PLGF cells had significantly higher percentage of M2MΦ (21.2 ± 2.8%) (p < 0.05, Fig. 2c). Moreover, PLGF alone was sufficiently to trigger this M2MΦ polarization (18.8 ± 2.1%) (Fig. 2b–c), which could be significantly abolished by a specific VEGFR1 antibody (1.2 ± 0.2%) (Fig. 2b–c). These data suggest that PLGF produced by the Hep2 cells is responsible for the adaptation of macrophage polarization in the co-culture system.

### M2MΦ expressed high levels of MMP9 and TGFβ1

**Figure 3:**
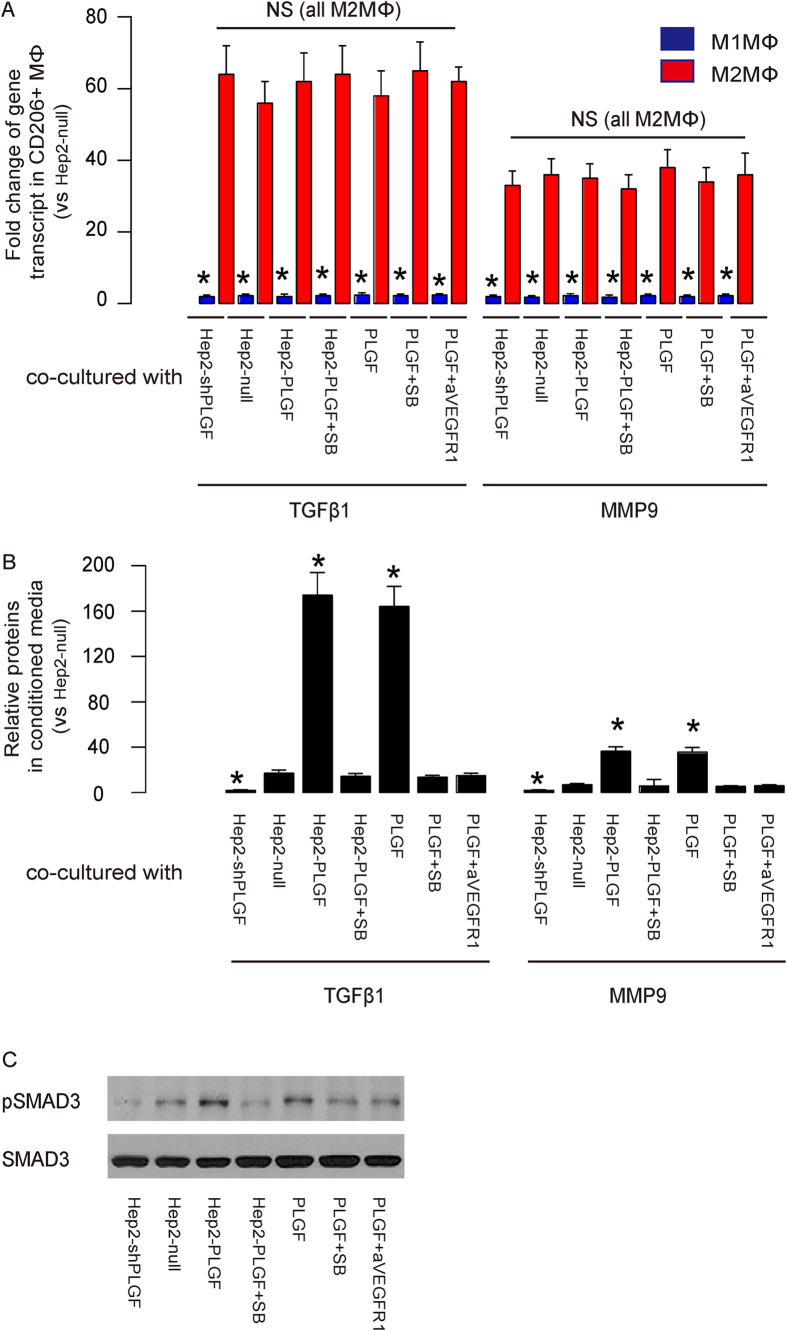
PLGF polarized macrophages to TAM to activate MMP9 and TGFβ1. (**a**) RT-qPCR for TGFβ1 and MMP9 in isolated M1MΦ and M2MΦ from the co-culture system. 2 × 10^4^ cells were used for RT-qPCR. (**b**) ELISA for TGFβ1 and MMP9 in the conditioned media from the co-culture system. (**c**) Western blot for SMAD3 and phosphorylated SMAD3 (pSMAD3). *: p < 0.05. NS: non-significant. N = 5. Statistic method: one-way ANOVA with a Bonferoni Correction.

Next, we examined the levels of MMP9 in the isolated M2MΦ, compared to those in M1MΦ, by RT-qPCR (Fig. 3a). We also examined the MMP9 levels in the conditioned media by ELISA (Fig. 3b). We found that the M2MΦ isolated from the co-culture system had nearly 40 fold higher MMP9, compared to the M1MΦ (Fig. 3a). There are no significant differences in the MMP9 levels either in the M2MΦ among different conditions, or in the M1MΦ among different conditions (Fig. 3a). These data suggest that M2MΦ induced by PLGF produced significant higher MMP9 than M1MΦ, consistent with our finding in the specimen from the LC patients (Fig. 1c,d). The analyses on the secreted protein in the conditioned media showed that PLGF significantly increased MMP9 secretion, and the levels of MMP9 seemed to be determined by the percentage or the number of M2MΦ (Fig. 3b).

Since M2MΦ have been shown to express high level of TGFβ1^30^,^34^,^35^, we were prompted to examine whether TGFβ receptor signaling may be responsible for the activation of MMP9 in M2MΦ. First, we analyzed TGFβ1 levels in the isolated macrophages after co-culture, and detected about 60 fold higher TGFβ1 transcripts in M2MΦ, compared to M1MΦ (Fig. 3a). Again, there are no significant differences in the TGFβ1 levels either in the M2MΦ among different conditions, or in the M1MΦ among different conditions (Fig. 3a). The secreted protein in the conditioned media reflected the percentage or the number of M2MΦ, showing that PLGF significantly increased TGFβ1 by macrophages (Fig. 3b).

Taken together, these data suggest that the PLGF induced polarization of macrophages to M2MΦ, which expressed high levels of both MMP9 and TGFβ1.

### PLGF-triggered activation of MMP9 in macrophages required TGFβ receptor signaling

In order to determine whether TGFβ receptor signaling is necessary for PLGF-triggered activation of MMP9 in macrophages, we added SB431542 (SB), a specific inhibitor for TGFβ receptor I^36^,^37^, to both the macrophages co-cultured with Hep2-PLGF and the macrophages with PLGF alone (Fig. 2b). The effects of SB were confirmed by examining the phosphorylation levels of a TGFβ receptor I downstream target SMAD3 by Western blot (Fig. 3c). We found that administration of SB completely abolished the effects of either Hep2-PLGF (0.8 ± 0.2% with SB vs 21.2 ± 2.8% without SB, p < 0.05) or PLGF (0.9 ± 0.2% with SB vs 18.8 ± 2.1% without SB, p < 0.05) on the polarization of macrophages (Fig. 2c). These data suggest that TGFβ receptor signaling is required for the PLGF-induced polarization of macrophages, and then is required for activation of MMP9 in macrophages for cancer metastasis in LC.

Consistent with the finding that the number of M2MΦ determined the secreted protein of MMP9 and TGFβ1 (Fig. 3a,b), our data show that SB significantly reduced PLGF-triggered production of both MMP9 and TGFβ1 in the conditioned media by macrophages (Fig. 3b). Thus, activation of MMP9 in macrophages by PLGF appears to require TGFβ receptor signaling.

### PLGF induced macrophage polarization *in vivo* in LC and promoted cancer growth

**Figure 4:**
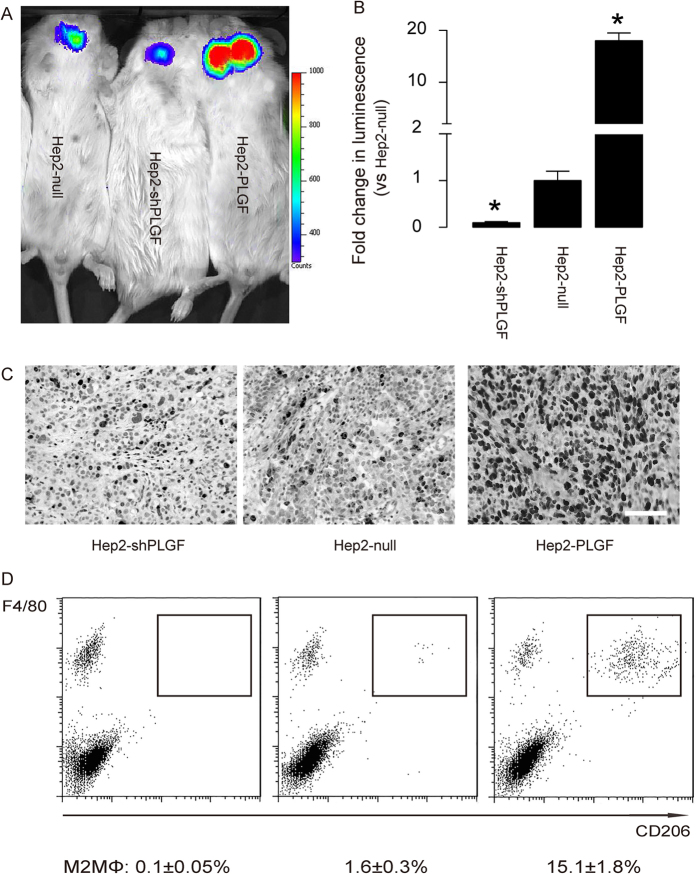
PLGF induced macrophage polarization *in vivo* in LC and promoted cancer growth. (**a-b**) Luciferase-carrying Hep2-shPLGF, Hep2-Null and Hep2-PLGF cells of 10^6^ each were implanted into NOD/SCID mice for 4 weeks before the tumor growth was quantified by luminescence levels, shown by representative images (**a**) and by quantification (**b**). (**c**) Representative images for Ki-67 staining. (**d**) A representative flow cytometry chart for analyzing digested implanted cancer. F4/80, a marker for macrophages. CD206, a marker for M2 macrophages (M2MΦ) or TAM. *: p < 0.05. N = 5. Statistic method: one-way ANOVA with a Bonferoni Correction. Scale bar is 50 μm.

To examine whether PLGF may similarly induce macrophage polarization *in vivo* in LC, we implanted luciferase-carrying Hep2-shPLGF, Hep2-Null and Hep2-PLGF cells of 10^6^ each into NOD/SCID mice. These mice were kept for another 4 weeks before the tumor growth was quantified by luminescence levels. We found significantly greater tumors in mice receiving Hep2-PLGF cells (p < 0.05), and significantly smaller tumors in mice receiving Hep2-shPLGF cells (p < 0.05), compared to those in mice receiving Hep2-Null cells, by presentative images (Fig. 4a), and by quantification (Fig. 4b). These data were further confirmed by immunstaining for Ki-67, a cell proliferation marker, in the tumor sections (Fig. 4c). Thus, these data suggest that PLGF promotes LC growth *in vivo*.

We then dissected out the implanted tumors, digested them, and then analyzed macrophages by flow cytometry. We found that overexpression of PLGF in LC significantly increased the percentage of the TAM/M2MΦ (15.1 ± 1.8%, p < 0.05), while inhibition of PLGF in LC significantly decreased the percentage of the TAM/M2MΦ (0.1 ± 0.05%, p < 0.05), compared to the control (1.6 ± 0.3%) (Fig. 4d). These data suggest that PLGF induced macrophage polarization *in vivo* in LC.

**Figure 5:**
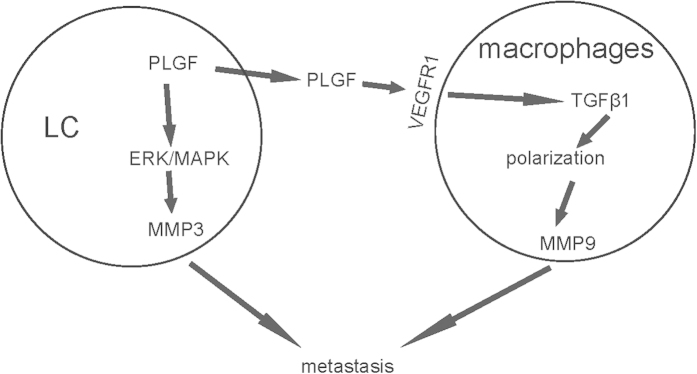
Schematic of the model. Together with our previous study, we demonstrates a model of how PLGF affects metastasis of LC. LC cells not only produce PLGF to activate MMP3 through an autologous way via ERK/MAPK signaling pathway, but also secretes PLGF to polarize macrophages into TAM in a TGFβ-receptor-signaling-dependent manner, which subsequently activates MMP9 in TAM. Both MMP3 from LC cells and MMP9 from TAM enhance cancer metastasis.

To summarize, together with our previous study^31^, we demonstrates a pivotal role of PLGF in the metastasis of LC, in which LC cells not only produce PLGF to activate MMP3 through an autologous way via ERK/MAPK signaling pathway^31^, but also secretes PLGF to polarize macrophages into TAM in a TGFβ-receptor-signaling-dependent manner, which subsequently activates MMP9 in TAM. Both MMP3 from LC cells and MMP9 from TAM enhance cancer metastasis (Fig. 5).

## Discussion

Understanding the molecular mechanism regulating the metastasis of LC may substantially improve its prognosis and therapy. During the invasion and migration of LC cells, they not only secrete angiogenetic molecules like VEGF and PLGF to increase capillary permeability and promote endothelial cell proliferation and survival, but also secrete MMPs to degrade extracellular matrix^16^,^17^,^18^,^19^,^20^,^21^,^22^. All these events are critical for cancer angiogenesis and metastasis. However, these processes are complicated and are regulated in a coordinate way by many factors and by the interaction of many cell types, including tumor cells, tumor endothelial cells, mesenchymal cells and inflammatory cells^14^,^15^. Of note, the role of TAM in tumorigenesis has been recently recognized, and their cross-talk with other types in the pathogenesis of various tumors has been reported^14^,^15^,^25^,^26^,^27^,^28^,^29^. However, a role of TAM in LC has been rarely addressed.

Our current study continued our previous work to address to the role of PLGF in the metastasis of LC. We have previously reported high MMP3 levels in LC cells through PLGF-mediated activation of ERK/MAPK^31^. Since the PLGF secreted by LC cells activated MMP3 in LC cells themselves, these data suggest that it may not be necessary for PLGF to be released out of the LC cells to activate the receptor signaling for activation of MMP3. In another word, PLGF may directly affect ERK/MAPK signaling pathway in LC cells for MMP3 activation, although the other possibility cannot be excluded. However, the regulation of MMP3 in LC cells appears to be through an autologous way, since PLGF is predominantly produced by LC cells. In the case of MMP9, here we clearly showed that the major sources for MMP9 were M2MΦ, rather than M1MΦ, or non-macrophages in LC tissue that included LC cells, tumor endothelial cells, mesenchymal cells, and non-macrophage leucocytes. Since macrophages are well-known to express VEGFR1, which is a receptor for VEGF-A and PLGF (For PLGF, VEGFR1 is the unique receptor, while VEGF-A also binds to another receptor called VEGFR2)^16^,^17^,^18^,^19^,^20^,^21^,^22^, we hypothesize that the PLGF in LC may affect macrophages through PLGF-VEGFR1 signaling.

In our previous study, we have prepared PLGF-modified human Hep2 LC cell lines. We have confirmed the adaptation of PLGF levels in these LC cells^31^. Here we used a co-culture system to examine the effect of these PLGF-modified LC cells on the polarization of bone-marrow derived macrophages and their production of MMP9. In the co-culture experiments, mouse macrophages were cultured together with human larynx carcinoma cells. To exclude a possibility that cells from different species may affect the results, we also performed a similar experiments using macrophages and carcinoma cells both from mouse. Since the results are very similar, a cross-species effect may be excluded.

To prove that the effect on macrophages was exactly resulted from the PLGF itself, but not from other factors from LC cells, we used PLGF alone as an additional control in a gain-of-function experiment. We found that PLGF was responsible for the macrophage polarization to a TAM phenotype, which produced high MMP9 and TGFβ1. Since TGFβ1 is a potential ligand for TGFβ receptor signaling, in which it binds to a TGFβ type II receptor, which subsequently phosphorylates a TGFβ type I receptor to trigger downstream signaling cascades^30^, we used a specific inhibitor for TGFβ type I receptor phosphorylation to determine whether TGFβ receptor signaling is required for macrophage polarization and MMP9 activation^30^. Indeed, this loss-of-function experiment confirmed our hypothesis. Moreover, the effects of PLGF were further confirmed by blocking its signaling through receptor inhibition by aVEGFR1. Thus, PLGF secreted by LC cells may activate the VEGFR1 on macrophages to polarize them into a TAM subtype, which releases TGFβ1 to further expand this polarization, and then TAM activate MMP9 to mediate LC metastasis. PLGF controls the polarization of macrophages, whereas the M2 macrophages from different sources had a similar levels of TGFβ1 and MMP9. These data suggest that the subtype determination of a macrophage may precede the activation of TGFβ1 and MMP9.

Since all these studies were done in vitro, we then analyzed the effect of PLGF from LC *in vivo*, since the effect of PLGF may be affected by systemic factors. However, our *in vivo* analyses on LC growth and macrophage polarization in LC tissue were consistent with our *in vitro* study, suggesting that the effect of PLGF on macrophages and the MMP9 activation by TAM also occurred *in vivo*. Moreover, PLGF significantly increased the growth of LC, which should be due to the increased MMP3 and MMP9 that destroy the extracellular matrix to allow the cells to grow, expand and invade periphery tissue. However, a direct effect of PLGF on LC growth may be present, since ERK/MAPK signaling is well-known for its role in regulating cell-cycle activators and inhibitors^38^. Moreover, TAM are well-known for their release of trophic factors, which may further enhance LC growth in a paracrine way^25^,^26^,^27^,^28^. Of note, macrophages are partially impaired in the NOD/SCID mice, which may attenuate the effects of PLGF on cancer cell growth and metastases, compared to wild-type mice.

To summarize, our study illustrates a novel model of the molecular mechanism underlying the angiogenesis and invasiveness of LC. Further delineation of the precise molecular mechanism that mediates the regulation of MMP9 in TAM that are polarized by PLGF may substantially improves our understanding of the controls for metastasis of LC.

## Additional Information

**How to cite this article**: Zhou, X. and Qi, Y. Larynx carcinoma regulates tumor-associated macrophages through PLGF signaling. *Sci. Rep.* **5**, 10071; doi: 10.1038/srep10071 (2015).
